# Application of Machine Learning Models for Early Detection and Accurate Classification of Type 2 Diabetes

**DOI:** 10.3390/diagnostics13142383

**Published:** 2023-07-15

**Authors:** Orlando Iparraguirre-Villanueva, Karina Espinola-Linares, Rosalynn Ornella Flores Castañeda, Michael Cabanillas-Carbonell

**Affiliations:** 1 Facultad de Ingeniería y Arquitectura, Universidad Autónoma del Perú, Lima 15842, Peru; oiparraguirre@ieee.org; 2 Facultad de Ingeniería, Universidad Tecnológica del Perú, Chimbote 02710, Peru; c24051@utp.edu.pe; 3 Facultad de Arquitectura e Ingeniería, Universidad César Vallejo, Lima 15314, Peru; rfloresc@ucv.edu.pe; 4 Facultad de Ingeniería, Universidad Privada del Norte, Lima 15083, Peru

**Keywords:** diabetes, machine learning, classification, modeling, analysis

## Abstract

Early detection of diabetes is essential to prevent serious complications in patients. The purpose of this work is to detect and classify type 2 diabetes in patients using machine learning (ML) models, and to select the most optimal model to predict the risk of diabetes. In this paper, five ML models, including K-nearest neighbor (K-NN), Bernoulli Naïve Bayes (BNB), decision tree (DT), logistic regression (LR), and support vector machine (SVM), are investigated to predict diabetic patients. A Kaggle-hosted Pima Indian dataset containing 768 patients with and without diabetes was used, including variables such as number of pregnancies the patient has had, blood glucose concentration, diastolic blood pressure, skinfold thickness, body insulin levels, body mass index (BMI), genetic background, diabetes in the family tree, age, and outcome (with/without diabetes). The results show that the K-NN and BNB models outperform the other models. The K-NN model obtained the best accuracy in detecting diabetes, with 79.6% accuracy, while the BNB model obtained 77.2% accuracy in detecting diabetes. Finally, it can be stated that the use of ML models for the early detection of diabetes is very promising.

## 1. Introduction

Diabetes occurs when blood sugar levels rise due to metabolic problems. This type of exposure can damage various organs and body systems, such as the heart, blood vessels, and eyes. It is important to note that these adverse effects are directly caused by hyperglycemia, which is elevated blood sugar levels. This is because the body has trouble controlling blood sugar levels or cannot properly use the insulin it produces [1]. The hormone insulin helps glucose reach and be available to the cells. Note that diabetes is divided into two main categories, type 1 and type 2. To fully understand type 1 diabetes, it is important to know that it is an autoimmune disease, which means that the body’s immune system constantly attacks and destroys insulin-producing cells.

Type 2 diabetes is characterized by problems with the proper use of insulin produced by the body due to factors related to an individual’s lifestyle [2]. During the last decade, a significant increase in the prevalence of type 2 diabetes has been observed in all countries of the world, regardless of socioeconomic status. It does not matter whether they are developed or developing countries [3]. In addition to causing blindness and kidney failure, diabetes can also lead to myocardial infarction, stroke, and lower limb amputations. Diabetics with poor glycemic control also have a significantly increased risk of cardiovascular disease and tuberculosis. The WHO/PAHO estimates that 6.7 million people will die from diabetes in 2022. Four out of five people with diabetes (81%) are from middle-income countries.

**Figure 1:**
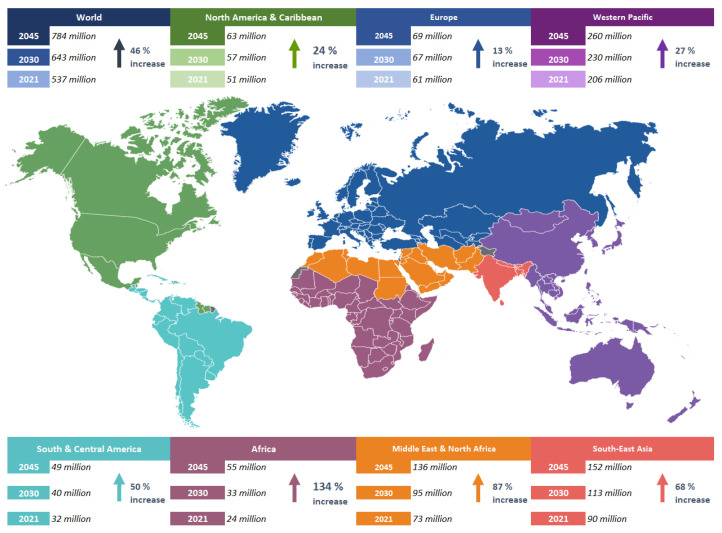
Number of diabetics in the world by region.

Adults are at high risk of diabetes, given that about 40% of them have not been diagnosed with the disease, and 90% of these people reside in middle-income countries. According to statistics, global spending on diabetes-related health care will reach USD 966 billion in 2021, an increase of 316% compared to the previous decade. Glucose intolerance is a problem that affects more than 541 million adults worldwide, according to the International Diabetes Federation (IDF). According to this statistic, about 10% of the U.S. population has a high likelihood of developing type 2 diabetes at some point in their lives. It is estimated that 68% of the adult population with diabetes live in countries with the highest rates of diabetes such as the United States of America [2,4]. The number of diabetics living in these countries was once 27.9 million. However, in the year 2021, approximately 537 million adults worldwide were counted as having diabetes, accounting for one in ten adult diabetics in the world. In a study published by the IDF, the number of diabetics worldwide was projected to reach 643 million by 2030 and 784 million by 2045. Currently, the Western Pacific region has the largest number of diabetic patients worldwide [5], as shown in Figure 1.

Artificial intelligence (AI), specifically, ML supervised learning, models are valuable tools that can help identify diabetes and manage this chronic disease. ML models have been shown to be excellent predictors of diabetes development. These models are based on information about a person’s medical history and other risk factors [6], as well as their genetic makeup [7]. ML models are also used to analyze medical images, such as CT scans and retinal scans, to detect early signs of diabetes and other related diseases [8]. ML is a new discipline where computer science and statistics come together to solve problems [9], developing breakthroughs with the ability to detect and classify gaps in patient care [10,11]. The purpose of these ML models is to contribute to improving the quality of patient care and reducing medical costs [12,13]. This work is useful in determining the risk factors responsible for the development of diabetes from clinical data and predicting prediabetes. Diabetes prevention can be achieved using a careful assessment of the patient’s socio-demographic and health conditions and implementing a personalized treatment plan that takes into account each patient’s specific risk factors and health conditions [14]. The aim of this work is to detect and classify type 2 diabetes in patients using ML models and to select the best classification model to predict the risk of diabetes. For the development of this work, the National Institute of Diabetes and Digestive and Kidney Diseases (NIH) dataset, hosted in Kaggle, is used.

## 2. Related Work

There are different works with ML for diabetes detection that used the Pima Indian dataset, which is a dataset composed of 9 characteristics and 768 patients [14,15,16,17]. For example, in [18], they implemented a model with ML using cross-validation to predict diabetes with noninvasive instruments, and they applied six ML models with different assessment metrics. As a result, the XG boost classifier obtained the best metric with 0.9013 in cross-validation, and the model with the lowest performance was SVM with 61.17%. The work [19] proposed a non-invasive methodology for the analysis of features extracted from tomographic images, and they used SVM as an ML classification model. The model performance using cross-validation reached an accuracy of 92.64% and a sensitivity of 91%. Similarly, Ref. [20] proposed a new ML-based framework for early prediction of type 2 diabetes mellitus. They used different ML techniques including the DT model, which achieved the highest performance rate with an accuracy of 0.9941, a precision of 0.99, a recall of 95.83%, a specificity of 99.11%, and an F1-score of 99.15%. Similarly, Ref. [21] used ML algorithms and neural networks (NNs) for diabetes prediction. The algorithms used included DT, random forest (RF), LR, Naïve Bayes (NB), KNN, and SVM, and they found that the LR and SVM algorithms obtained better performances in terms of accuracy for diabetes prediction. The article by Ref. [22] differs from the previous ones in that they decided to implement a model with ML to predict the blood insulin level, reaching an accuracy of 80%. In [23], using the DT model and the random tree (RT) model, they were able to create a data classifier that could detect whether a patient was diabetic or not based on the collected data. In recent years, diabetes has been considered the deadliest disease and is on the rise. Therefore, Ref. [24] examined several ML algorithms, including KNN, NB, DT, and multilayer perceptron (MP), to find the best classifier that can be used as a tool in healthcare facilities to improve decision-making. Also, in [25], they examined different supervised learning techniques to predict more accurately whether a patient has diabetes or not. The level of accuracy achieved with the RF model was superior to the others. The authors of [26] implemented a retrospective study using an ML algorithm that predicted diabetes within 5 years after diagnosis, and the model achieved an accuracy rate of 82%. Similarly, the work [27] conducted a comparative study with different classifiers to predict diabetes more accurately using the classifiers MP, DT, KNN, RF. The accuracies achieved were 0.7760 using the MP model, 0.7607 using DT, 0.7878 using KNN, and 0.798 using RF, meaning that the RF model achieved the best performance in that work. The work [28] proposed diagnostic software based on ML methods for diabetes identification. For the proposal, they used DT, AdaBoost (AB), and RF algorithms. The results showed that the proposed method effectively detected diabetes and can be implemented in an e-healthcare environment. In [29], the authors developed a method to classify diabetes, for which they used a Gaussian process (GP)-based classification technique, where the GP model achieved 81.97% accuracy performance. ML models, deep learning (DL) models, convolutional neural networks (CNNs), and NNs have contributed significantly to medical sciences. For example, in [30], they proposed an automated knowledge model for the purpose of identifying diabetes, for which they trained two types of neural networks: DL and CNN. As a result, with DL networks, they did not obtain the expected accuracy rates due to the existence of a hidden layer, while with CNNs, the accuracy rate improved significantly since they were able to quantify the characteristics of different classes more accurately.

## 3. Materials and Methods

**Figure 2:**
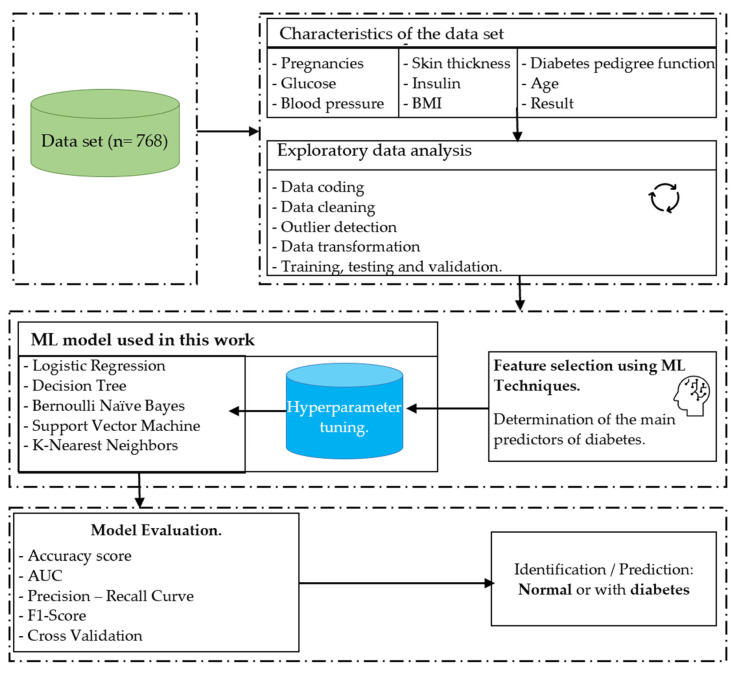
ML model development process. The process shows database extraction and analysis, variable selection, training, and selection of the best model based on its performance.

This section describes the methods and materials used in this study. It is arranged as follows: (1) description of the data source; (2) description of the variables included in the dataset and the tools used; (3) description of the ML models (LR, DT, BNB, SVM, K-NN) used in this work; (4) data processing: description of the techniques used for data cleaning and preparation; (5) exploratory data analysis, taking into account the techniques used to explore and visualize the data; (6) data modeling: training of the ML models; and (7) model validation: description of the techniques used to validate and evaluate the models. The model development process is shown in Figure 2.

### 3.1. Models

#### 3.1.1. Logistic Regression

An LR model is a type of ML model that is often used to predict the probability of an event as a function of a predictor variable, such as yes/no, true/false, and success/failure [31]. LR uses a mathematical function called the sigmoid function to estimate the probability that a dependent variable takes a particular value. In Ref. [32], the function is mathematically represented as logit (*p*) = log (*p*/(1 − *p*)) = $\beta_{0}$ + $\beta_{1\times 1}$ + $\;\beta_{2\times 2}$ + … + $\beta_{p\times p}$. LR is a statistical model that uses the logit function expressed in Equation (1) [33].

(1) $$f\left(x\right)=\frac{1}{1+e^{-x}}$$

#### 3.1.2. Decision Tree

A DT model is an ML model used for predictive analysis. Its application consists of several steps. (1) First, the attribute that best separates the data into two different groups is chosen; (2) once an attribute is selected, the dataset is divided into two subsets according to the value of that attribute; and (3) this process is repeated for each subset until it reaches a certain degree of consistency [34]. DT uses a series of recursive distributions to build tree structures to predict target variables from new observations.

#### 3.1.3. Bernoulli Naïve Bayes

A BNB model is an ML-based classification algorithm, which interprets features as binary variables that are autonomous from each other [35]. It is mainly used in text analysis applications to classify items into different categories [36]. A model is trained using discrete data, where the features are only in binary form. The model calculates the probability of each feature in each class and uses these probabilities to predict the class for a new observation [37]. The Bernoulli distribution is presented in Equation (2).

(2) $$P\left[X=x\right]=P^{x}{\left(1-P\right)}^{1-x}\;x=0;\;x=1$$

#### 3.1.4. Support Vector Machine

An SVM model is an algorithm used to solve classification and regression problems. The model finds the hyperplane that maximizes the distance between the two closest classes and the distance between samples [38]. In the case of two classes or two dimensions, the hyperplane is represented using the following Equation (3).

(3) $$\beta_{0}+\beta_{1\times 1}+\beta_{2\times 2}=0$$

where, for parameters $\beta_{0}$, $\beta_{1},$ and $\beta_{2}$, in this case, all pairs of *x* = (*x*_1_, *x*_2_) are hyperplane points. Mathematically, for p dimensions, this can be generalized as in Equation (4).

(4) $$\beta_{0}+\beta_{1\times 1}+\beta_{2\times 2}+.\ldots .\ldots +\beta_{p\times p}=0$$

#### 3.1.5. K-Nearest Neighbor

Using a K-NN model, one can solve classification problems as well as regression problems using machine learning. As part of the classification process, the model calculates how far away a sample, from all other samples in the dataset, is from the sample that needs to be classified [39]. Using the K nearest samples to the sample to be classified, the class or the numerical value of the sample to be classified is determined [40]. The model uses different types of distances to calculate the closeness between samples, and the value of K can be adjusted to improve the performance of the model [41]. In the following Equations (5)–(7), the distance metrics are represented.

(5) $$\mathrm{Euclidian}\;\mathrm{distance}\;d\left(x,y\right)=\sqrt{\sum_{i=1}^{n}{\left(y_{i}-x_{i}\right)}^{2}}$$

(6) $$\mathrm{Manhattan}\;\mathrm{distance}\;d\left(x,y\right)=\left(\sum_{i=1}^{m}\lfloor x_{i}-y_{i}\rfloor \right)$$

(7) $$\mathrm{Minkowski}\;\mathrm{distance}\;d\left(x,y\right)\;={\left(\sum_{i=1}^{n}\lceil x_{i}-y_{i}\rceil \right)}^{1/p}$$

### 3.2. Development of the Case Study

This section provides a detailed explanation of the development of the case study. The Pima Indian diabetes dataset, provided by the NIH and hosted in Kaggle, is used to make a diagnostic prediction about the presence or absence of diabetes in a patient, based on certain criteria embedded in the dataset. This dataset consists of several variables related to medical predictors and one variable related to outcomes. Several variables contribute to the prediction of diabetes in a patient, such as the number of pregnancies a woman has had and her glucose level, diastolic blood pressure, skinfold thickness, insulin levels, body mass index (BMI), genetic history of diabetes in the family, age, and outcome. It is very important to point out that, in this study, synthetic oversampling of minorities (SMOTE) was used. This is because the dataset is not large in volume. Therefore, the entire dataset was used as input, but only a small percentage of cases belong to the minority group. Unlike the existing minority cases, the algorithm does not simply duplicate them. Instead, by sampling the feature space of all target classes and their nearest neighbors, it not only generates copies of the existing minority cases but artificially increases the number. In addition to generating original examples, the algorithm also combines the features of the cases to create original examples. With this approach, it is possible to increase the number of features available for each class, as well as to make the samples more meaningful.

The prediction of diabetes using ML models is performed using binary classification, where the objective is to identify whether a patient has diabetes or not, based on the different characteristics in the dataset. In this work, the following ML models were used: LR, DT, BNB, SVM, and K-NN. It is important to note that this study used the Pima Indian dataset to train these ML models. The case study analysis used these data to train the model and adjust the parameters so that it can accurately predict diabetes in new people. Also, it is necessary to validate the model with test data to assess its accuracy.

#### 3.2.1. Dataset Processing

This section starts with loading the dataset from Kaggle, including a total of 768 patient records with 9 characteristics (number of pregnancies in women, glucose level (amount of sugar circulating in the blood), diastolic blood pressure, thickness of skin folds, insulin level, body mass index, genetic history of diabetes, age, and diabetes result (yes/no)). For this step, the pandas library, read_csv(), was used, as shown in Table 1.

Next, the exploratory data analysis was performed using histograms and libraries such as drop for the cleaning process, which was of significant help since it allowed for identifying characteristics that have zero values and, in turn, replace them with some value. After applying the histogram, the dataset consists of 268 diabetics and 500 individuals without diabetes. Next, we proceeded to check the statistical values of the dataset. For example, the number of observations; the mean of the values, which provides a measure of the centrality of the data; the standard deviation, which indicates the variability or spread of the values; and the minimum and maximum values were considered to understand the range of the data. In addition, lower quartile values (Q1), the median, and upper quartile values (Q3) provide information about the distribution of the data in percentile terms. The results are shown in Table 2.

**Figure 3:**
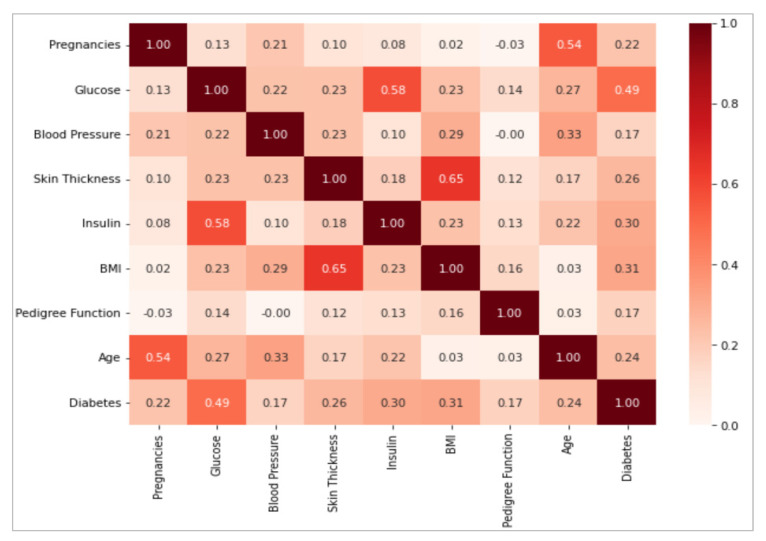
Correlation matrix of variables in the dataset.

One of the main tasks in the detection and classification of diabetes using ML models is to analyze how the variables in the dataset are related to each other, which uses data analysis techniques and software tools. For this task, we used the Python language to import the dataset, calculate the correlation matrix, and perform the technique in the correlation analysis. It is essential to emphasize that the existence of a correlation between two variables does not necessarily imply a causal relationship between them. That is, the fact that two characteristics are correlated does not imply that one is the direct cause of the other. In this paper, we used correlation analyses to examine the association between variables. Correlation is measured on a scale ranging from −1 to 1, where the negative value (−1) indicates a perfect negative correlation and the positive value (1) indicates a perfect positive correlation. When the correlation is equal to zero (0), this indicates that there is no linear association between the variables. The interpretation of the correlation is important to establish the strength and direction of the relationship between the variables. If we look at Figure 3, we observe that most of the characteristics present a low correlation, with values below 0.5. However, some values that exceed this threshold stand out, such as the case between age and pregnancies, where it is evident that the number of pregnancies increases as age advances and stops after a certain age. Likewise, a significant correlation is found between glucose and insulin, where an increase in glucose levels is associated with a higher probability of diabetes diagnosis. For glucose and diabetes: the higher the glucose level, the higher the amount of insulin required to regulate it. In addition, there is a relationship between BMI and body fat: the higher the BMI, the higher the percentage of fat in the patient.

**Figure 4:**
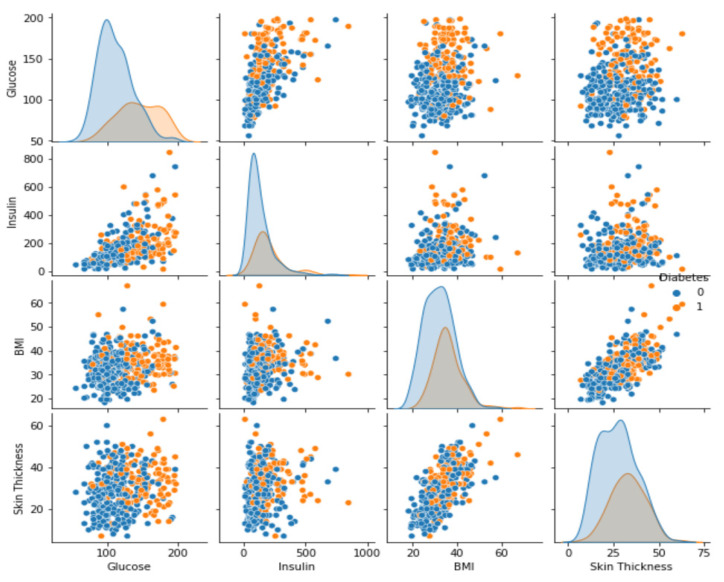
Correlation of variables using scatter plots.

In Figure 3, it is evident that the correlation between variables is not very strong. Therefore, we next analyzed the variables using a scatter plot. This graphical tool helps to present the relationship between two variables in a quantitative way with the purpose of determining how correlated the variables are. To perform the scatter analysis, four variables were selected from the dataset, including skin thickness, body mass index, insulin, and glucose, with the objective of seeing if there is a relationship between the four variables. Figure 4 shows a direct correlation between glucose levels, insulin, BMI, and skin thickness. Therefore, these are the variables that will play a very important role in the prediction of diabetes. Therefore, it can be concluded that an increase in the value of one variable leads to an increase in the value of the other variable, indicating a positive relationship between the two.

After finishing the exploratory analysis of the dataset, the next step was to run the training. This process started by dividing the data into a ratio of 80% for the training set and 20% for the test set. For this, we used the library Sklearn.Model_selection.train_test_split() since this library allows us to perform the analysis by simply specifying test sizes. As in the exploratory analysis, it was observed that the dataset contains null or missing data. Therefore, we proceeded to correct them by eliminating them from the dataset, for which we used libraries SimpleImputer() and impute.fit_transform(train, test).

Next, we normalized the data. Normalization of the dataset is a crucial step, as it involves organizing and scaling each of the features according to the standard deviation. This allows all features to be on the same scale. To perform feature normalization, we used the main Python libraries including StandardScaler(prm), StandardScaler.fit_transform(train_prm, test_prm), and data_train_normalized(prm). Since the dataset is not large enough, two techniques, SMOTE and principal component analysis (PCA), were applied to artificially sample and avoid overfitting. This measure was used because the dataset for this work consisted of only 768 values. These techniques helped to balance class imbalance since class imbalance can generate asymmetries in training and result in overfitting. To evaluate the effectiveness of these techniques, the accuracy obtained using the original dataset was compared with that obtained using the sampled dataset.

**Figure 5:**
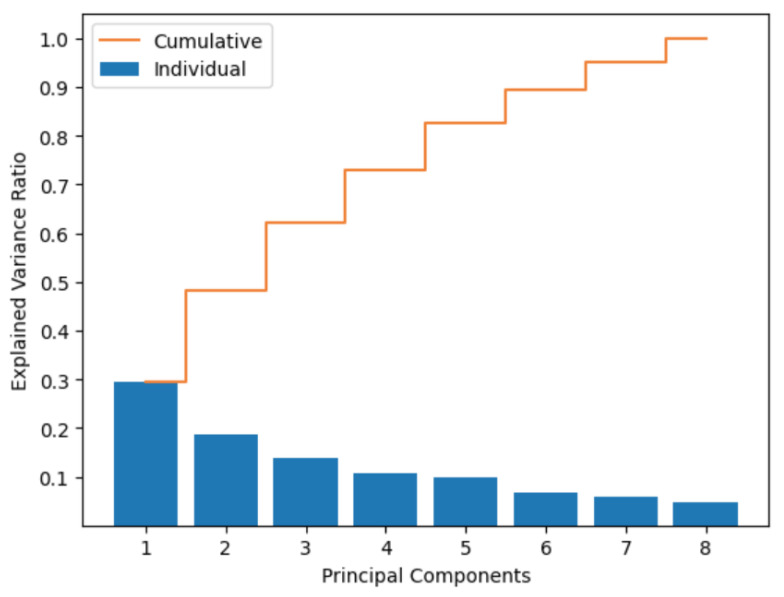
Principal component analysis.

PCA is an unsupervised ML technique used to reduce the complexity of datasets. This technique consists of projecting the original dataset into a lower dimensional space composed of a set of principal components. These principal components represent the directions in which the dataset has the highest variability. In other words, PCA makes it possible to identify and retain only the most relevant features in the dataset, eliminating those that provide little information. This facilitates an understanding and analysis of the data by reducing the number of variables in the dataset without losing critical information. The technique consists of the following steps (1) normalization of the dataset; (2) calculation of the covariance matrix of the sample; (3) decomposition of the sample to obtain eigenvalues and eigenvectors; (4) construction of the projection matrix with vectors; and (5) PCA transformation to obtain the reduced dataset. To obtain the number of reduced functions, we identified the amount of variation represented by each principal component. Figure 5 shows that with the higher number of retained principal components, there is higher conserved variance and a higher number of functions after applying PCA.

**Figure 6:**
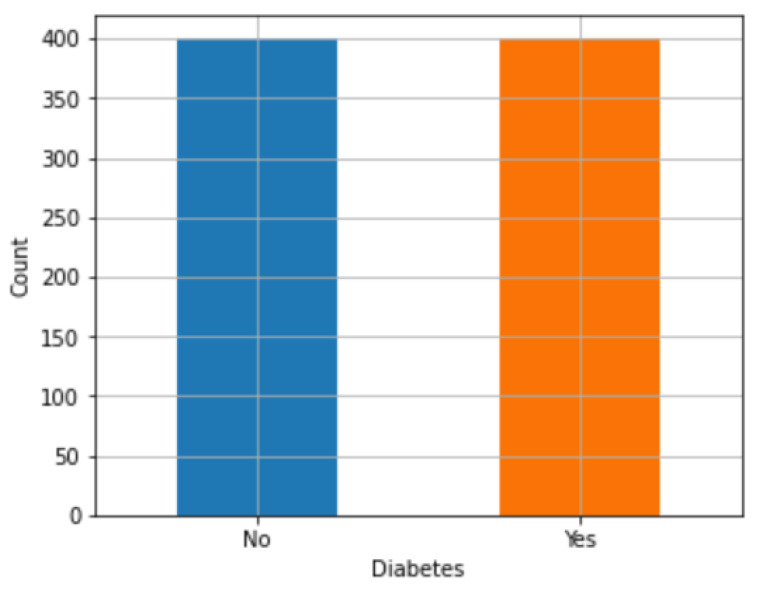
Application of SMOTE to the dataset.

SMOTE. The SMOTE technique is very useful for addressing imbalance problems in a dataset. This strategy was used with the purpose of increasing the efficiency of ML models by increasing the number of samples belonging to the underrepresented class. In the Python implementation, the packages imbalanced-learn(), scikit-learn(), among others, were used. These packages offer functions that allow for applying the SMOTE technique to a dataset and fitting ML models such as LR, DT, BNB, SVM, and K-NN using the synthetic samples generated with SMOTE. The process for generating synthetic samples using SMOTE consists of the following steps: First, the difference between the minority class sample and one of its K-NN was calculated. This difference was then multiplied by a random number between 0 and 1 and added to the minority class sample. Then, a point on the line segment between two data points was randomly selected, and a new sample of the minority class was synthesized. In this way, the representation of the minority class in the data set increased, allowing a better balance, and avoiding possible biases in the ML model. This method helped to balance the two types of diabetes. For example, Figure 6 shows that after applying SMOTE, there are 50% of people with diabetes and 50% without diabetes.

#### 3.2.2. Training and Evaluation of Models

**Figure 7:**
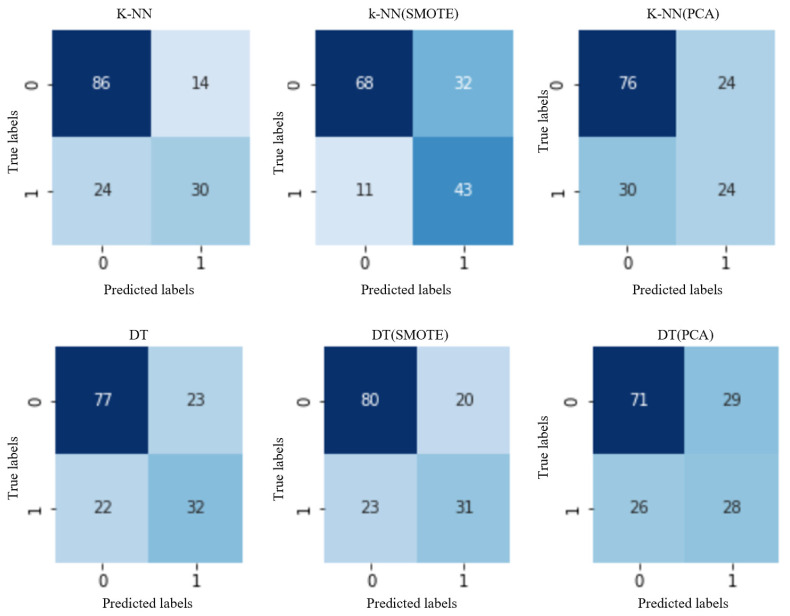
Confusion matrices for the K-NN and DT models.

Training the ML models involves feeding data to the algorithm so that it can learn and recognize patterns and make accurate predictions. For each of the classifiers, three different models were fitted: one using the original data model, one using the SMOTE-balanced dataset, and one using the PCA-reduced dataset [42]. In the process of selecting the optimal parameters for each classifier, the cross-validation technique was used, which allowed for estimating the hyperparameters that produced the best results in a more efficient way than simply using the training dataset. Cross-validation consists of dividing the dataset into smaller subsets and running the model several times, using different subsets for training and evaluation in each iteration. In this way, several estimates of the model’s capacity were obtained, allowing for a more rigorous selection of the parameters that produced the best performance. For this analysis, the 5-fold algorithm was applied, and as a result, the average validation score for each parameter setting was obtained. Since the dataset was small, we decided to apply two validation techniques. First, for the original dataset and the PCA-reduced dataset, the F1-score was selected, which takes into account class imbalance and the precision-recovery trade-off. Then, for the oversampling data set obtained using SMOTE, the precision was selected because the algorithm balances the samples of the two classes. After fitting the models identified using cross-validation, the predictions for the test set data samples were evaluated. To evaluate the performance of the ML model, several indicators were used, which are defined as (1) true positives (TP); in this work, those patients with positive diagnoses of diabetes were classified as positive samples; (2) true negatives; in this work, negative samples were classified to those patients who were not diagnosed with diabetes; (3) false positives were samples misclassified as positive; and (4) false negatives were samples misclassified as negative. The results are shown in Figure 7.

Figure 7 shows the confusion matrices for the first two models, K-NN and DT. For example, the K-NN model for the original dataset obtained 86 samples classified as patients with a positive diagnosis of diabetes and 32 samples classified as negative samples, which represent those patients who were not diagnosed with diabetes. Applying the SMOTE technique to the dataset, we obtained 80 samples classified as patients with a positive diagnosis of diabetes and 31 negative samples, representing those patients who were not diagnosed with diabetes. And applying the PCA technique to the dataset, 71 samples were classified as patients with a positive diagnosis of diabetes, and 28 negative samples were classified are those patients who were not diagnosed with diabetes. The same procedure was followed for the other models used in this work.

**Figure 8:**
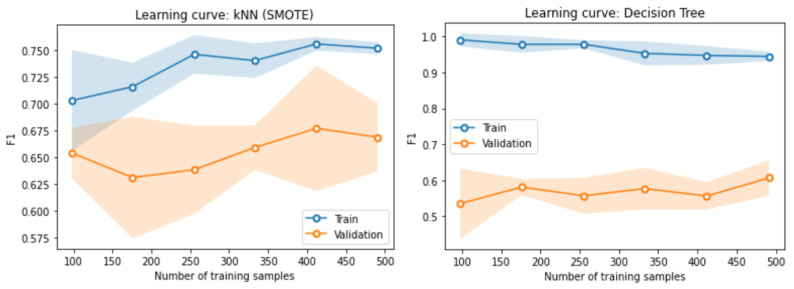
Learning curve for the K-NN and DT models.

Once a model is trained, it is crucial to perform an evaluation of its performance to ensure its accuracy and efficiency. The procedure carried out was as follows: 20% of the dataset was set aside for testing the model. Various evaluation metrics, such as confusion matrix, accuracy, F1-score, precision, and recall, were used to assess its performance. Figure 8 shows the learning curve for the first two models, K-NN and DT, which indicates that the training and validation scores increased with the number of samples in the training set. However, using more than 400 samples did not improve the performance of the K-NN model since both scores remained almost constant. Similarly, for the DT model, the scores remained almost constant from the first samples.

## 4. Results

After the training process for the K-NN, DT, LR, BNB, and SVM models using the original dataset, SMOTE, and PCA, the original dataset consisted of 768 patients with and without diabetes and 9 features for training. After balancing the data, where 80% and 20% were used for training and validation, respectively, we proceeded with training. The purpose was to detect and classify patients with type 2 diabetes using ML models and select the best classification model to predict the risk of diabetes. To determine the performance of the classification models, different metrics were used, such as the learning curve, as shown in Figure 8. Metrics such as the F1-score, accuracy, precision, and recall were also used. As can be seen, the only model that obtained acceptable results was K-NN when applying the SMOTE technique to the original dataset, which reached 79.6% accuracy. Applying PCA this model reached 44.4%, and with the original dataset, it reached 55.6%. The next model that achieved good results was BNB when applying SMOTE, which reached 77.2% accuracy. When applying the PCA technique, the BNB model achieved 59.70% performance, and when training with the original dataset, it achieved 66.2% accuracy. Therefore, according to the results, the two best models to identify and classify type 2 diabetes using an ML model are K-NN and BNB. There was not much difference between the results of the two models, as shown in Table 3.

Finally, the two models that obtained the best results are shown in Table 4. The two models were ML and supervised learning models. Each has strengths and weaknesses, and the choice between the two depends on the specific characteristics of the classification problem being addressed. Since the dataset used in this study was small, the SMOTE technique had to be applied to artificially increase the dataset so that the training would be as realistic as possible. In this way, these two models achieved acceptable results, with 79.6% and 77.2% accuracy, respectively.

## 5. Discussion

The detection and classification of diabetes is a problem for data science and medical science. There are many ML algorithms that are used to address this problem. In this paper, we used five of the most popular algorithms used to identify and classify binary problems such as diabetes including K-NN, DT, LR, BNB, and SVM. All five models are very useful for diabetes classification, but each has its own advantages and disadvantages. For example, K-NN is simple and easy to implement but can be sensitive to outlier data points. On the other hand, BNB is less sensitive to outlier data points and performs well on large and noisy datasets. The DT, LR, and SVM models behave in much the same way, and they are widely used algorithms for diabetes classification, each with its advantages and disadvantages. DTs are easy to understand and fit, LR is simple and fast, and SVMs are very accurate in classifying nonlinear data. The results achieved in this work, mainly with the K-NN and BNB models, are acceptable, as shown in Table 4. The K-NN model achieved an accuracy of 79.6%; this result is similar to that obtained in [21] where they used this algorithm and neural networks to classify diabetes, obtaining an accuracy of 88.6%. However, in [24], the K-NN model reached an accuracy of 86% when used to diagnose and classify diabetes using ML algorithms. The results depend directly on the dataset and its characteristics. In a similar way, these results were also found with the BNB model, which also achieved a very good result of 77.2% accuracy. However, this result is significantly different from that achieved in [19] since in that work, it was used to classify peripheral arterial disease in patients with type 2 diabetes using ML models, and the model achieved a performance of 92% accuracy and a sensitivity of 91.80%. The difference in accuracy results is mainly due to the dataset with which it is processed and the characteristics it includes. The DT and SVM models did not obtain the expected results, only reaching 63% and 71.7% accuracy, and these results are below the threshold. However, the DT model used in [18] achieved a performance of 90.8% accuracy. Meanwhile, the SVM model used in [18] only achieved a performance of 57.4% accuracy. This does not mean that ML models are not efficient classifiers, rather, the results basically depend on the characteristics of the dataset. Finally, the LR model achieved a performance of 72.7% accuracy. This result is related to that obtained in the work [22], where they used this model to predict diabetes based on ML, achieving a performance of 75% accuracy. In the different works reviewed, it is evident that the LR model has not obtained significant results, probably due to the simplicity of the algorithm at the time of training and prediction. In addition, there are factors that affect the results.

The strengths of this work for detecting and classifying type 2 diabetes basically lie in the following: (1) Accuracy: ML models are able to identify complex patterns in large datasets. (2) Scalability: ML models can also handle large volumes of data efficiently. (3) Flexibility: ML models can be used to predict diabetes in different populations. (4) Speed: ML models generate very fast predictions; hence, they can help to make decisions in real-time.

## 6. Conclusions

Predicting diabetes remains one of the most challenging areas within the field of medical engineering. After performing this work, which detected and classified type 2diabetes using the ML models K-NN, DT, BNB, LR, and SVM, the following conclusions were reached. ML models are useful tools for detecting diabetes in patients. (1) K-NN(SMOTE) and BNB(SMMOTE) models had superior performance compared to the DT, LR, and SVM models. (2) The K-NN(SMOTE) model achieved the best performance, with an accuracy of 79.6% in detecting diabetes. (3) The BNB(SMOTE) model was the second-best performing model, with an accuracy of 77.2% in diabetes detection. (4) The DT model showed an accuracy of 63% in diabetes detection. (5) The LR(SMOTE) model also showed an accuracy of 72.7% in diabetes detection. (6) The SVM(SMOTE) model had an accuracy of 71.7% in the detection of diabetes. It is important to point out that the accuracy of the ML models was affected by the amount of data used for training, so the SMOTE and PCA techniques had to be applied to artificially increase the volume of the dataset. In general terms, ML models have enormous potential for the detection of diabetes in patients. However, limitations need to be considered.

In this work, there were certain limitations such as (1) the 768 patients in the dataset were women over 21 years and had indigenous heritage and (2) the volume of the dataset was relatively small for detection and classification to work efficiently. Therefore, the SMOTE and PCA techniques were used, which are synthetic minority oversampling statistical techniques to increase the number of cases in a dataset in a balanced manner.

## Figures and Tables

**Table 1:** Diastolic blood pressure.

Column	Not Empty Count	Dtype
Number of pregnancies in women	768 (not empty)	long64
Glucose level (amount of sugar in the blood)	768 (not empty)	long64
Diastolic blood pressure	768 (not empty)	long64
Thickness of skin folds	768 (not empty)	long64
Insulin levels	768 (not empty)	long64
BMI	768 (not empty)	Float64
Genetic history of diabetes	768 (not empty)	Float64
Age	768 (not empty)	long64
Diabetes result (yes/no)	768 (not empty)	long64
dtypes: float64 (2), long64 (7)		

**Table 2:** Checking Statistical Values.

	Pregnancies	Glucose	Blood Pressure	Skin Thickness	Insulin	BMI	Diabetes Pedigree Function	Age	Diabetes
Quantity	768	763	733	541	394	757	768	768	768
mean	3.85	121.69	72.41	29.15	155.55	32.46	0.472	33.24	0.35
std	3.37	30.54	12.38	10.48	118.78	6.92	0.333	11.76	0.48
minimum	0	44	24	7	14	18.2	0.081	21	0.00
25%	1	99	64	22	76.25	27.5	0.254	24	0.00
50%	3	117	72	29	125	32.3	0.367	29	0.00
75%	6	141	80	36	190	36.6	0.653	41	1
maximum	17	199	122	99	846	67.1	2.24	81	1

**Table 3:** Training Results for the ML Models.

	F1-Score	Accuracy	Precision	Recall
K-NN				
SMOTE	0.667	0.721	0.573	0.796
KNN	0.612	0.753	0.682	0.556
PCA	0.471	0.649	0.500	0.444
DT				
DT	0.602	0.708	0.576	0.630
SMOTE	0.590	0.721	0.608	0.574
PCA	0423	0.610	0.440	0.407
LR				
SMOTE	0.555	0.694	0.597	0.727
LR	0.513	0.698	0.612	0.672
PCA	0.485	0.594	0.530	0.648
BNB				
SMOTE	0.677	0.692	0.479	0.772
BNB	0.387	0.461	0.528	0.662
PCA	0.529	0.539	0.582	0.597
SVM				
SMOTE	0.56	0.701	0.588	0.717
SVM	0.53	0.670	0.618	0.689
PCA	0.461	0.628	0.539	0.462

**Table 4:** Comparing the two Best Models.

Best Performing Models				
	F1-Score	Accuracy	Precision	Recall
K-NN (SMOTE)	0.667	0.721	0.573	0.796
BNB (SMOTE)	0.677	0.692	0.479	0.772

## Data Availability

Not applicable.
